# Protective Role of Sirtuin3 (SIRT3) in Oxidative Stress Mediated by Hepatitis B Virus X Protein Expression

**DOI:** 10.1371/journal.pone.0150961

**Published:** 2016-03-07

**Authors:** Ji-Hua Ren, Xiang Chen, Li Zhou, Na-Na Tao, Hong-Zhong Zhou, Bo Liu, Wan-Yu Li, Ai-Long Huang, Juan Chen

**Affiliations:** 1 Key Laboratory of Molecular Biology for Infectious Diseases (Ministry of Education), Institute for Viral Hepatitis, Department of Infectious Diseases, The Second Affiliated Hospital, Chongqing Medical University, Chongqing, China; 2 Collaborative Innovation Center for Diagnosis and Treatment of Infectious Diseases, Zhejiang University, Zhejiang, China; 3 Department of Epidemiology, School of Public Health and Management, Chongqing Medical University, Chongqing, China; Drexel University College of Medicine, UNITED STATES

## Abstract

**Background/Aim:**

The hepatitis B virus (HBV) infection is accompanied by the induction of oxidative stress, especially mediated by HBV X protein (HBx). Oxidative stress has been implicated in a series of pathological states, such as DNA damage, cell survival and apoptosis. However, the host factor by which cells protect themselves under this oxidative stress is poorly understood.

**Methodology/Principal Findings:**

In this study, we first confirmed that HBV infection significantly induced oxidative stress. Moreover, viral protein HBx plays a major role in the oxidative stress induced by HBV. Importantly, we found that mitochondrial protein SIRT3 overexpression could decrease reactive oxygen species (ROS) induced by HBx while SIRT3 knockdown increased HBx-induced ROS. Importantly, SIRT3 overexpression abolished oxidative damage of HBx-expressing cells as evidenced by γH_2_AX and AP sites measurements. In contrast, SIRT3 knockdown promoted HBx-induced oxidative damage. In addition, we also observed that oxidant H_2_O_2_ markedly promoted HBV replication while the antioxidant N-acetyl-L-cysteine (NAC) inhibited HBV replication. Significantly, SIRT3 overexpression inhibited HBV replication by reducing cellular ROS level.

**Conclusions/Significance:**

Collectively, these data suggest HBx expression induces oxidative stress, which promotes cellular oxidative damage and viral replication during HBV pathogenesis. Mitochondrial protein SIRT3 protected HBx expressing-cells from oxidative damage and inhibited HBV replication possibly by decreased cellular ROS level. These studies shed new light on the physiological significance of SIRT3 on HBx-induced oxidative stress, which can contribute to the liver pathogenesis.

## Introduction

Human HBV infection is a public health problem which affects nearly 350 million people worldwide [1]. Many studies have shown that HBV infection could induce oxidative stress by using HBV-expressing cell model and HBV transgenic mouse model. Patients with HBV infection also show increased oxidative stress and oxidative damage. Excess reactive oxygen species (ROS) produced from oxidative stress could damage cellular molecules like lipids, protein and DNA during chronic HBV infection and finally leads to development of liver disease. Therefore, identification and characterization of the host factors which could protect hepatocyte from oxidative damage will provide valuable information for the development of anti-HBV therapeutics.

Sirtuins are generally known as a conserved family of class III nicotinamide adenine dinucleotide (NAD) dependent histone deacetylases (HDACs). Seven members of the sirtuin family have been identified in mammals (SIRT1-7). Among SIRT1-7, SIRT3 is a major mitochondrial deacetylase that targets no less than 20% of the proteome located in mitochondrial [2]. Intriguingly, it deacetylates and activates several mitochondrial proteins that involved in mitochondrial oxidative metabolism and energy production, such as subunits of complex II and V of the electron transport chain [3–6]. Recently, SIRT3 has been also identified as a stress responsive deacetylase and plays an important role in protecting cells under stress conditions. SIRT3 could attenuate the effect of oxidative stress on several different cell lines [2, 7–9]. In addition, the SIRT3-catalyzed deacetylation of 8-oxoguanine-DNA glycosylase 1 (OGG1) protects mitochondrial DNA from oxidative damage and prevents apoptotic cell death under oxidative stress [10]. These studies highlight the significance of SIRT3 to protect cells from oxidative damage.

In this study, we focused on the role of SIRT3 in HBV-induced oxidative stress. We found that SIRT3 protected HBx expressing-cells from oxidative damage and inhibited HBV replication possibly by decreasing cellular ROS level. These studies shed new light on the physiological significance of SIRT3 on HBx-induced oxidative stress which can contribute to the liver pathogenesis.

## Materials and Methods

### Plasmids and antibodies

pCH9/3091 was obtained from Lin Lan (The Third Military Medical University, Chongqing, China). pCH9 was constructed by digesting the HBV genome in the pCH9/3091 and ligating with T4 DNA ligase (Takara, Kusatsu, Shiga, Japan). The MUT HBV plasmid was constructed by site-directed mutagenesis of pCH9/3091 (as the wild-type HBV, WT HBV) via introduction of a stop codon at the beginning of the HBx gene. Site-directed mutagenesis was carried out by PCR amplification of the WT HBV. The primer carried a C-to-T mutation at nt 1397. This mutation results in a stop codon mutation in the HBx gene (codon 8) without affecting the polymerase gene product. pcDNA3.1-Flag-SIRT3 was obtained from ADDGENE (Cambridge, MA, USA). SiRNA targeted SIRT3 was obtained from Invitrogen (Carslbad, CA, USA). Rabbit anti-SIRT3 monoclonal antibody, anti-γ-H_2_AX monoclonal antibody and anti-PRDX1 monoclonal antibody were obtained from Cell Signaling Technology (CST, Danvers, MA, USA). Rabbit anti-Flag monoclonal antibody was obtained from Sigma (St Louis, MO, USA). Rabbit anti-β-actin monoclonal antibody was obtained from Santa Cruz Biotechnology (Santa Cruz, CA, USA). Tetracycline, tert-butyl hydroperoxide (tBOOH) and H_2_O_2_ were obtained from Sigma. NAC (S0077) was obtained from Beyotime (Haimen, Jiangsu, China).

### Cell culture and transfection

Huh-7 cells were maintained in Dulbecco’s modified Eagle medium (DMEM, gibco by Life Technologies, Carlsbad, CA, USA) containing 10% fetal bovine serum. HepG2 cells were maintained in modified Eagle medium (MEM, CORNING, Manassas, VA, USA) containing 10% fetal bovine serum. HepG2.2.15 and HepAD38 (a HepG2 cell line that produces HBV when it is grown in the absence of tetracycline) were maintained in MEM containing 10% fetal bovine serum and 400 μg/ml G418. In addition, HepAD38 cells were grown in the presence of 0.3 μg/ml tetracycline. All cells were maintained in an incubator containing 5% CO_2_ at 37°C. Transfection was carried out using X-treme GENE HP DNA Transfection Reagent (Roche, Basle, Switzerland).

### Determination of cellular Reactive oxygen species (ROS)

Cells were plated on a 6-well plate. ROS generation was measured using Image iT LIVE Green Reactive Oxygen Species Detection Kit (Invitrogen) according to the manufacturer’s protocol. In detail, when cells are ready, gently wash cells once with warm PBS buffer. Then apply a sufficient amount of 25 mM carboxy-H_2_DCFDA working solution to cover the cells adhering to the plate. Incubate for 30 minutes at 37°C, protected from light. After that gently wash cells three times in warm PBS and add some more PBS buffer before imaging the cells immediately under fluorescence microscopy. In addition, Quantitative analysis of cellular ROS levels was carried out by flow cytometry. The fluorescence of 10,000 cells was collected for each of three independent experiments.

### Determination of mitochondrial ROS

Cells were plated on a 6-well plate. Mitochondrial ROS was detected by MitoSOX^TM^ Red (Molecular Probes) (Invitrogen) according to the manufacturer’s protocol. In detail, cells were washed once with pre-warmed PBS. Then apply a 1 ml of the 5 μM MitoSOX™ reagent working solution to cover the cells adhering to coverslips. Incubate for 10 minutes at 37°C, protected from light. Then wash cells gently three times with warm PBS buffer and imaging the cells immediately under fluorescence microscopy. In addition, Quantitative analysis of mitochondrial ROS levels was carried out by flow cytometry. The fluorescence of 10,000 cells was collected for each of three independent experiments.

### Determination of GSH/GSSG

Cells were plated on a 6-well plate and transferred to a 96-well plate after transfection for one day. GSH/GSSG ration assays were performed by GSH/GSSG-Glo™ Assay (Promega, Madison, WI, USA) according to the optimized protocol provided in the kit.

### AP sites assay

Genomic DNA of cells transfected with indicated plasmids was extracted with Wizard® Genomic DNA Purification Kit (Promega). AP sites in the genomic DNA were measured by DNA Damage Quantification Kit (DOJINDO, Kumamoto, Kyushu, Japan) according to the manufacturer’s protocol. Briefly, the genomic DNA was labeled by Aldehyde Reactive Probe (ARP). Then the standard ARP DNA and purified ARP-treated sample DNA was fixed on the 96 well plates with DNA Binding Solution. The number of AP sites in the sample DNA was determined by the biotin-avidin-peroxidase assay.

### Cell viability assay

Cell viability was measured by CellTiter 96® AQueous One Solution Cell Proliferation Assay (MTS, promega) according to the manufacturer's protocol. Cells transfected with indicated plasmids were seeded in 96-well plate. The cells under treatment with incremental doses of tBOOH were incubated for 72 h before the addition of MTS solution. Finally, the absorbance was read at 490 nm using a microplate reader (BIO-TEK, Winooski, VT, USA).

### Apoptosis analysis

Apoptosis was determined by cell apoptosis kit (Invitrogen) according to the instruction manual. Briefly, 100 μl annexin-binding buffer was added to 1 × 10^5^ cells, and the cells were incubated with 5 μl Alexa Fluor® 488 annexin V and 1 μl 100 μg/mL PI working solution for 15 minutes. After the incubation period, 400 μl annexin-binding buffer was added to the sample and mixed gently. As soon as possible, the stained cells were analyzed by flow cytometry.

### Immunofluorescence

For immunofluorescence, cells were plated on glass coverslips. At 72 h post transfection, cells were fixed with 4% paraformaldehyde in PBS for 15 min, and permeabilized with 0.1% TritonX-100 in PBS for 10 min at room temperature. After blocking with 1% BSA in PBS for 45 min, the cells were incubated with the anti-γH_2_AX (Dilution1:100) overnight at 4°C. The cells were then washed sequentially in PBS and incubated with Alexa Fluor 555 anti-rabbit secondary antibody for 2 h at room temperature. Finally, the nuclei of the cells were stained with 4’, 6-Diamidino-2-Phenylindole Dihydrochlorie (DAPI) for 10 min at room temperature. The stained cells were visualized on a TCS-SP5X microscope (Leica, Milton Keynes, UK)

### Western blot analysis

Protein concentration was determined by using a BCA protein assay (Bio-Rad, Hercules, California, USA). Equivalent amounts of protein (30 μg per lane) were loaded and separated by SDS-PAGE, and transferred onto a nitrocellulose membrane (GE Healthcare, Buckinghamshire, UK). The membranes were blocked with 5% nonfat milk for 1 h and incubated with the relevant primary antibodies overnight at 4°C on an orbital shaker. After that the membranes were washed and incubated with secondary antibody for 2 h at room temperature. Blots were developed by using ECL western blot reagents.

### HBV replicative intermediates purification and analysis

HBV replicative intermediates were obtained as follows. To exclude possibility that the change of the HBV copies was due to different cell number, HBV replicative intermediates were extracted in the same number of cells. In detail, the same number of cells was disrupted in 500 μl lysis buffer (10 mM Tris-HCl [pH 8.0], 1 mM EDTA, 1% NP-40, 2% sucrose) at 37°C for 15 min. After centrifugation, the supernatant was treated with 40 U of DNase I/ml and 10 mM MgCl_2_ for 4 h for at 37°C to remove the extra-capsid nuclear acid, and then mixed with 200 μl of 35% polyethylene glycol 8000 containing 1.5 M NaCl on ice for 1 h. After centrifugation, the pellet contained viral nucleocapsids was incubated with digestion buffer containing 0.5 mg/ml proteinase K (Takara), 0.5% sodium dodecyl sulfate (SDS), 150 mM NaCl, 25 mM Tris-HCl (pH 8.0), and 10 mM EDTA. HBV replicative intermediates in the digestion mixture were isolated by phenol-chloroform (1:1) extraction and ethanol precipitation. Then the HBV replicative intermediates were dissolved in TE (10 mM Tris-HCl [pH 8.0], 1mM EDTA) buffer. The quantification of HBV copies was performed by FastStart Essential DNA Green MasterMix (Roche) using the sense primer: CCTAGTAGTCAGTTATGTCAAC and the anti-sense primer: TCTATAAGCTGGAGGAGTGCGA. The pCH9/HBV1.1 plasmid at different concentrations (3 × 10^7^, 3 × 10^6^, 3 × 10^5^, 3 × 10^4^, 3 × 10^3^ copies/μl) was used as a template to create the standard curve. All the samples were cycled once 95°C for 3 min, then 10 cycles of 94°C for 15 s, 65°C for 30 s, and 72°C for 20 s, finally, 30 cycles of 94°C for 15 s, 55°C for 30 s, and 72°C for 20 s. The HBV DNA copy number was calculated according to the standard curve. For southern blot, HBV replicative intermediates were loaded onto 0.9% agarose gels, blotted onto nylon membranes, After UV cross-linking and prehybridization, the membrane was hybridized with digoxigenin-labeled HBV-specific probe generated with DIG-High Prime (Roche). The signal was detected by exposing on an X-ray film.

### Statistical analysis

Results are expressed as means ± the SD. Differences between two groups were evaluated by two-tailed Student's t tests, differences between multiple groups were evaluated by one-way ANOVA. P value < 0.05 was considered statistically significant.

All statistical analyses were conducted using the statistical program SPSS version 17.0.

## Results

### Hepatitis B virus replication induces oxidative stress

**Fig 1:**
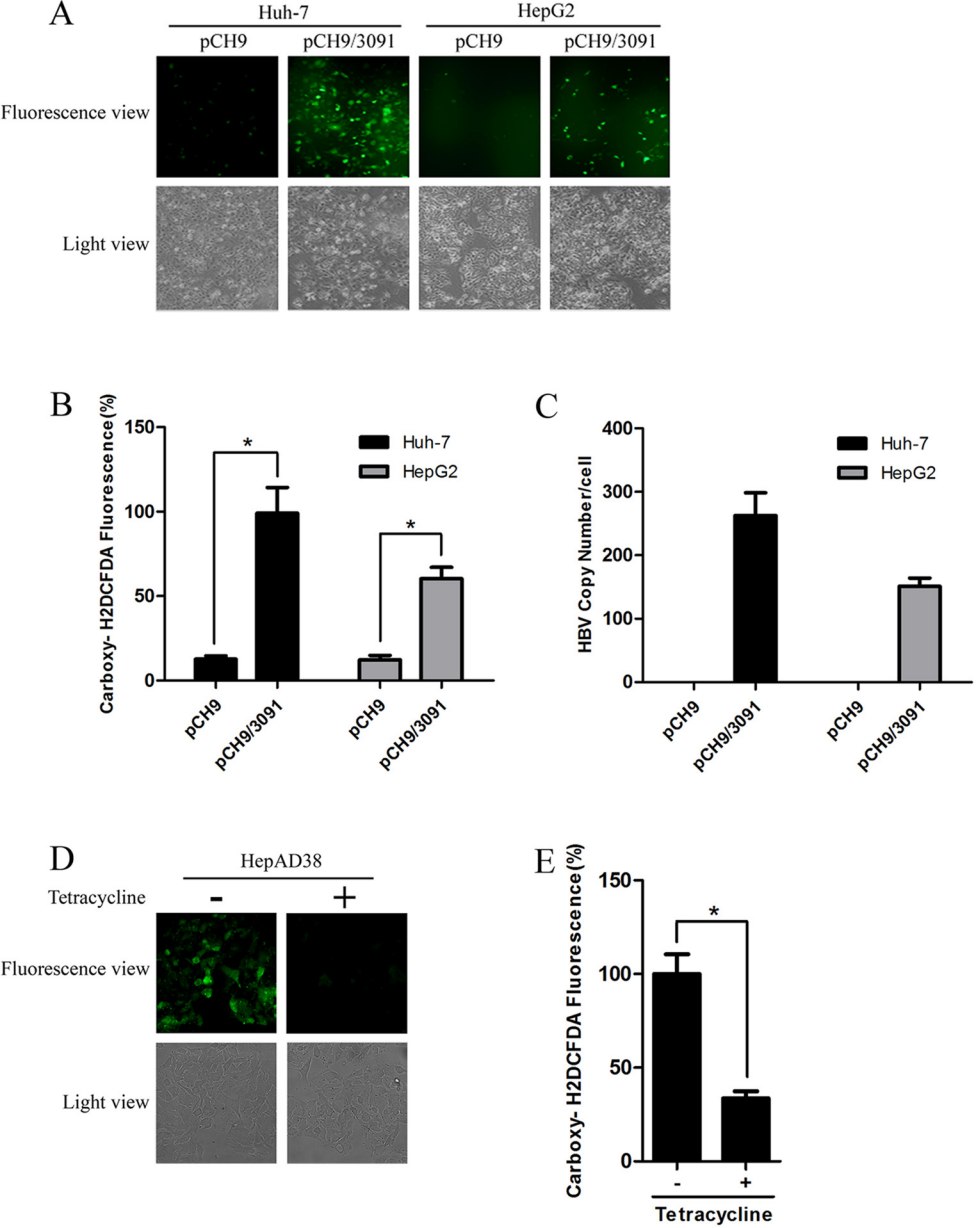
HBV replication induces oxidative stress. (A) ROS levels in Huh-7 and HepG2 cells transfected with pCH9 or pCH9/3091 were examined by carboxy- H2DCFDA fluorescence. Magnification, ×100. (B) The ROS level in Huh-7 and HepG2 cells transfected with pCH9 or pCH9/3091 was analyzed by flow cytometry. *, p<0.01. (C) The HBV replicative intermediates in Huh-7 and HepG2 cells transfected with pCH9 or pCH9/3091 were measured by real-time PCR. The results were expressed as the number of HBV DNA copies per cells. *, p<0.05. (D) ROS levels in HepAD38 cells cultured in medium with tetracycline (No HBV replication) or without tetracycline (HBV replication) were examined by carboxy-H2DCFDA fluorescence. Magnification, × 400. (E) The ROS levels in HepAD38 cells cultured with or without tetracycline were detected by flow cytometry. *, p<0.01.

Many studies have shown that HBV could induce oxidative stress using HBV transgenic mice or HBV DNA-transfected cells [11, 12]. To observe whether cellular oxidative stress was directly induced by HBV replication in our cell model, HepG2 and Huh-7 cells were transfected with the pCH9/3091 plasmid containing the HBV genome or with vector pCH9. Cellular ROS level was determined by both immunofluorescence and Carboxy-2, 7-dichlorofluorescein diacetate (carboxy-H2DCFDA) staining methods. HBV-expressing cells exhibited a greater percentage of ROS-positive cells than their control cells (Fig 1A). Flow cytometry assay confirmed that increased ROS level was observed in HBV-expressing cells (Fig 1B). HBV replication level was verified by real-time PCR (Fig 1C). In order to further confirm the oxidative stress caused by HBV replication, the ROS level was examined in HepAD38 cells (A HepG2 cell line stably expressing HBV under control of tetracycline). Consistently, increased ROS level in HepAD38 cells cultured without tetracycline (HBV replication) was observed compared with HepAD38 cells with tetracycline (No HBV replication) (Fig 1D and 1E). Taken together, these data suggest that HBV replication induces oxidative stress.

### Viral protein HBV X protein plays a major role in oxidative stress

**Fig 2:**
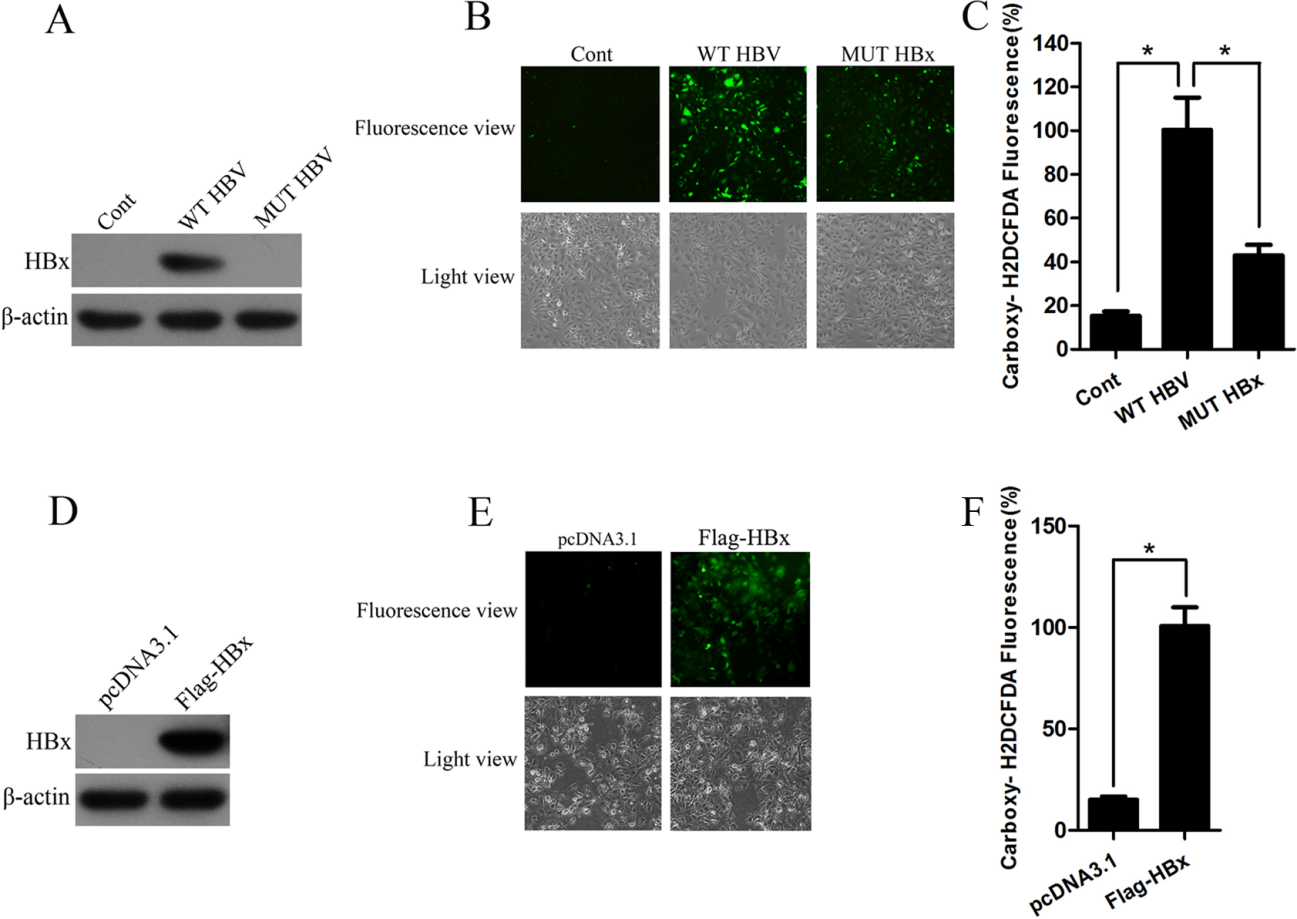
HBx induces oxidative stress. (A) The expression of HBx in Huh-7 cells transfected with HBV expressing plasmid (WT HBV) or plasmid expressing HBV genome with HBx mutation (MUT HBV) was measured by western blot analysis. β-actin was used as a loading control. (B-C) ROS levels in Huh-7 cells transfected with WT HBV or plasmid expressing MUT HBV were examined by carboxy-H2DCFDA fluorescence (B) or flow cytometry (C). *, p<0.01 (D) Western blot analyzed the HBx proteins in cells transfected with pcDNA3.1 or Flag-HBx. β-actin was used as a loading control. (E) ROS levels in Huh-7 cells transfected with pcDNA3.1 or Flag-HBx were examined by carboxy-H2DCFDA fluorescence. Magnification, ×100. (F) ROS levels in Huh-7 cells transfected with pcDNA3.1 or Flag-HBx were examined by flow cytometry. *, p<0.01.

HBx is a key regulator of HBV and exerts pleiotropic activity on cellular functions. To identify the role of HBx in HBV-induced oxidative stress, human hepatoma Huh-7 cells were transfected with vector pcDNA3.1, HBV expressing plasmid (WT HBV) or plasmid expressing HBV genome with HBx mutation (MUT HBV). Western blot analysis confirmed that Huh-7 cells transfected with WT HBV expressed HBx protein while no HBx protein was observed in cells transfected with MUT HBV (Fig 2A). Strikingly, Huh-7 cells expressing wild–type HBV genome showed increased ROS level compared with cells transfected vector, whereas the ROS level was remarkably decreased in cells transfected HBx-mutated HBV genome (Fig 2B). Consistently, flow cytometry also showed the ROS production induced by HBV infection was abolished in cells transfected with HBx-mutated HBV genome (Fig 2C). To further determine whether HBx is required for HBV-induced cellular oxidative stress, Huh-7 cells were transfected with pcDNA3.1 or Flag-HBx. Western blot analysis showed that HBx was markedly overexpressed (Fig 2D). Compared with cells transfected with control vector pcDNA3.1, Huh-7 cells ectopically expressing HBx showed increased level of ROS nearly 5 folds (Fig 2E and 2F). These data suggest HBx could directly increase the ROS level.

### SIRT3 attenuates the oxidative stress induced by HBx expression

**Fig 3:**
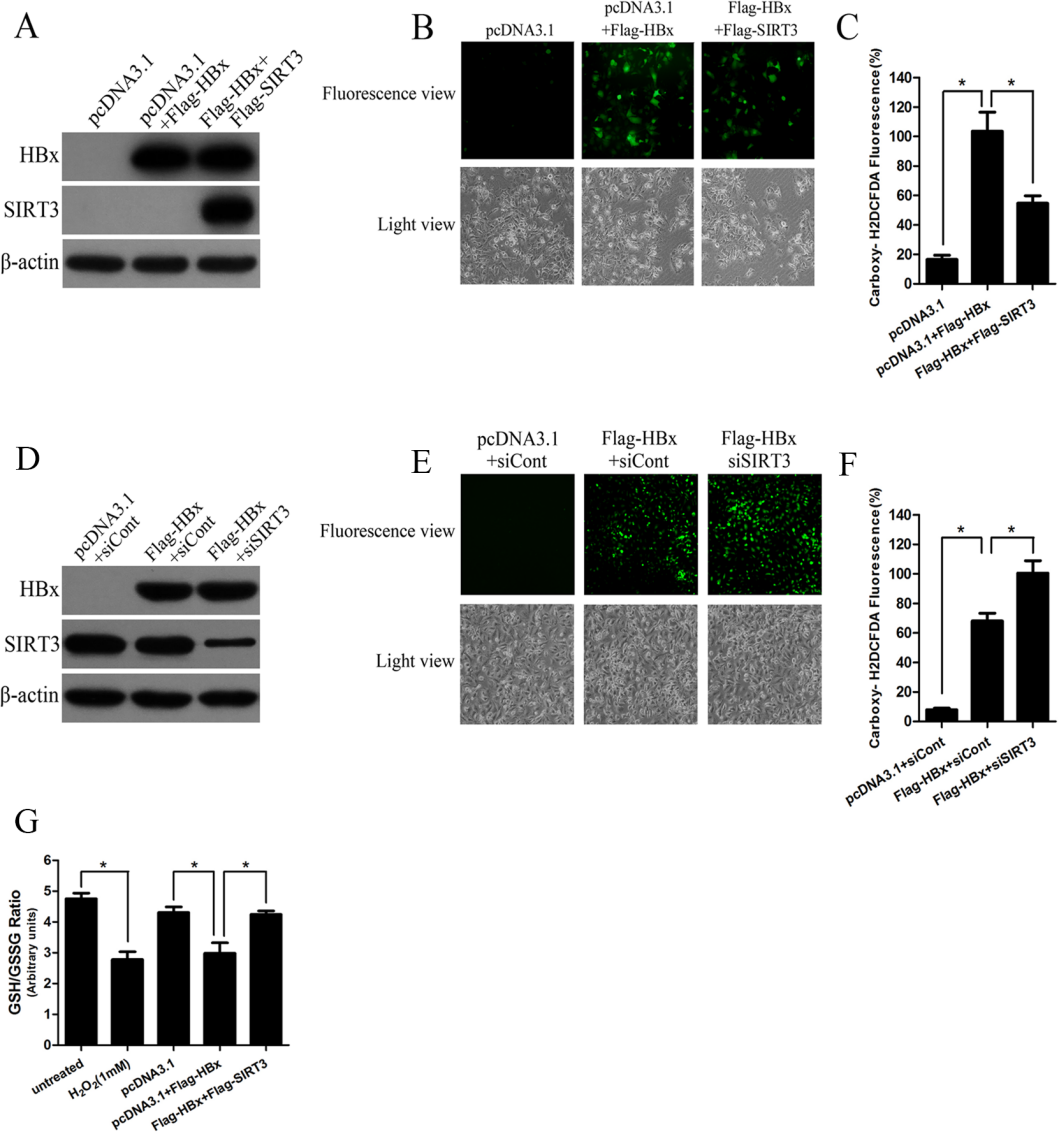
SIRT3 attenuates the oxidative stress induced by HBx expression. (A) Western blot analyzed HBx and SIRT3 in cells transfected with indicated plasmids. β-actin was used as a loading control. (B-C) ROS levels in Huh-7 cells transfected with indicated plasmids were examined by carboxy-H2DCFDA fluorescence (B) or flow cytometry (C). Magnification, ×100. *, p<0.01. (D)The expression of HBx and SIRT3 in cells transfected with indicated plasmids and siRNA. β-actin was used as a loading control. (E-F) ROS levels in Huh-7 cells transfected with indicated plasmids and siRNA were examined by carboxy-H2DCFDA fluorescence (E) or flow cytometry (F). Magnification, ×100. *, p<0.05. (G) The GSH/GSSG ratio in Huh-7 cells treated with 1 mM H_2_O_2_ or transfected with indicated plasmids was analyzed by GSH/GSSG-Glo™ Assay. *, p<0.01.

Oxidative stress is associated with mitochondrial dysfunction in many diseases. SIRT3 functions mainly as the primary mitochondrial deacetylase that modulates mitochondrial metabolic and oxidative stress regulatory pathways. Our group have identified that HBV replication, especially HBx expression, could downregulate SIRT3 expression (Data not shown in this manuscript). Therefore, we investigate whether SIRT3 plays an important role in HBx-induced oxidative stress. Huh-7 cells were cotransfected with plasmid pcDNA3.1, Flag-SIRT3 or Flag-HBx and the efficiency of SIRT3 and HBx overexpression was measured by western blot (Fig 3A). Interestingly, immunofluorescence analysis showed that SIRT3-overexpressing cells exhibited less percentage of ROS-positive cells (Fig 3B). Consistently, flow cytometry found that SIRT3 overexpression resulted in a significant inhibition of oxidative stress induced by HBx expression (Fig 3C). To investigate whether HBx could induce mitochondrial ROS, cells transfected with indicated plasmids were stained with MitoSOX^TM^ Red. Both immunofluorescence analysis and flow cytometry showed that HBx also induced mitochondrial ROS while SIRT3 overexpression attenuated increased mitochondrial ROS level in HBx-expressing cells (S1A and S1B Fig). To further prove the role of SIRT3 in HBx-induced oxidative stress, we inhibited SIRT3 expression by siRNA targeted SIRT3 (siSIRT3). The knockdown efficiency of SIRT3 was determined by western blot analysis (Fig 3D). Consistently, gene silencing of SIRT3 in HBx-expressing cells significantly increased ROS level compared to cells ectopically expressing HBx as evidenced by immunofluorescence analysis (Fig 3E) and flow cytometry (Fig 3F). To prove the specific protective effect of SIRT3 in HBx-induced oxidative stress, we also overexpressed Peroxiredoxin 1 (PRDX1), which is a member of the peroxiredoxin family of antioxidant enzymes, in HBx-expressing cells. Unlike SIRT3, PRDX1 overexpression has no effect on HBx-induced ROS level (S2A–S2C Fig). On the other hand, as an important index of oxidative stress, cells GSH/GSSG was also monitored and H_2_O_2_ treatment was included as positive control. The GSH/GSSG ratio in H_2_O_2_-treated cells was decreased when compared with the untreated controls (Fig 3G). Similarly, HBx expression caused a dramatically fall in the GSH/GSSG ratio, whereas SIRT3 overexpression resulted in increased GSH/GSSG ratio in HBx-expressing cells (Fig 3G). To summarize, these data reveal SIRT3 attenuates HBx-induced oxidative stress.

### SIRT3 increases resistance of HBx-overexpressing cell to oxidant damage

**Fig 4:**
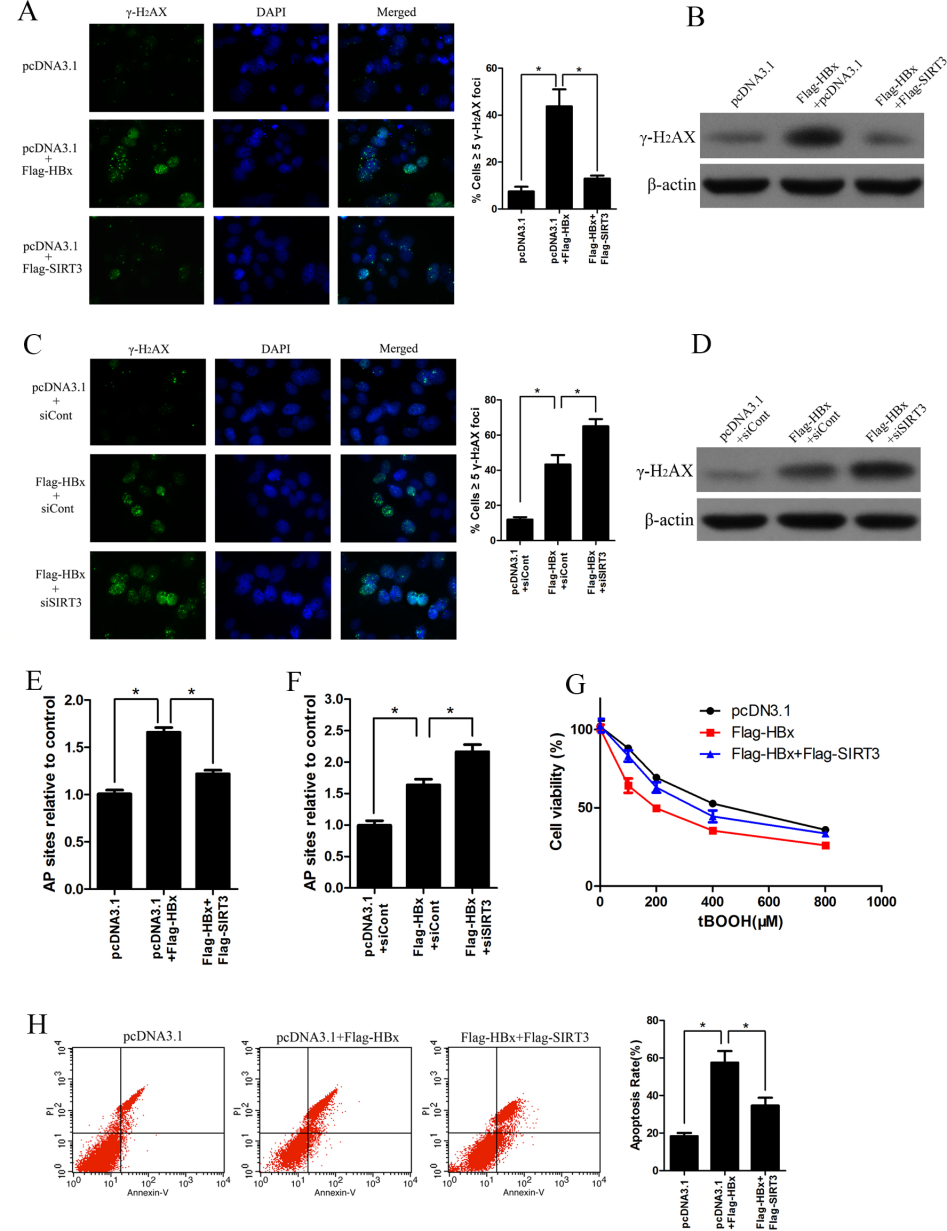
SIRT3 increases resistance of HBx-overexpressing cell to oxidant damage. (A) Immunofluorescence of histone H_2_AX phosphorylation (γH_2_AX) in Huh-7 cells transfected with indicated plasmids for 3 days. Nuclei were stained with DAPI (left panel). Quantification of γH_2_AX foci (right panel). *, p<0.01. (B) Western blot analysis of γH_2_AX in Huh-7 cells transfected with indicated plasmids for 3 days. β-actin was used as a loading control. (C) Immunofluorescence of γH_2_AX in Huh-7 cells transfected with indicated plasmids and siRNA for 3 days. Nuclei were stained with DAPI (left panel). Quantification of γH_2_AX foci (right panel). *, p<0.05. (D) Western blot analysis of γH_2_AX in Huh-7 cells transfected with indicated plasmids and siRNA for 3 days. β-actin was used as a loading control. (E) AP sites numbers in Huh-7 cells transfected with indicated plasmids for 3 d were estimated by DNA Damage Quantification Kit, *, p<0.001. (F) AP sites numbers in Huh-7 cells transfected with indicated plasmids and siRNA for 3 d were estimated by DNA Damage Quantification Kit, *, p<0.01. (G) The cell viability in Huh-7 cells transfected with indicated plasmids under tBOOH treatment were measured by MTS assay. (H) Cell apoptosis in Huh-7 cells transfected with indicated plasmids under 100 μM tBOOH treated were measured by flow cytometry with Annexin V/PI. *, p<0.05.

Excess ROS generated in oxidative stress could damage lipids, protein or DNA, which finally induces oxidative DNA damage leading to increased chromosomal aberrations associated with cell transformation. The oxidatively induced DNA damage associated with ROS typically are apurinic/apyrimidinic (abasic) DNA sites, oxidized purines and pyrimidines, single strand (SSBs) and double strand (DSB) DNA breaks. To investigate the role of SIRT3 in oxidative damage induced by HBx, we first examined histone H_2_AX phosphorylation (γH_2_AX) which is a DSB DNA breaks marker by using immunofluorescence. HBx overexpression induced more γH_2_AX foci in cells compared with vector-expressing cells, suggesting HBx could induce oxidative damage in Huh-7 cells (Fig 4A). Moreover, ectopic expression of SIRT3 attenuated the γH_2_AX foci in HBx-expressing cells (Fig 4A). In addition, western blot analysis further confirmed that SIRT3 antagonized H_2_AX phosphorylation by HBx (Fig 4B). On the other hand, we also investigated the role of SIRT3 knockdown in the γH_2_AX formation caused by HBx expressing. Both immunofluorescence and western blot analysis showed that the SIRT3 knockdown increased the γH2AX foci in HBx-expressing cells (Fig 4C and 4D). AP sites are one of the major types of damage generated by ROS. Therefore, we measured the number of AP sites in DNA extracted from Huh-7 cells transfected with indicated plasmids. HBx induced higher level of AP sites while SIRT3 overexpression abolished the enhancement of AP sites in HBx-expressing cells (Fig 4E). In contrast, SIRT3 knockdown increased the level of AP sites in HBx expressing cells (Fig 4F). Oxidative stress is a well-known factor that could induce cell death [13, 14]. We next tested whether SIRT3 overexpression could protect Huh-7 cells expressing HBx against oxidative damage. Huh-7 cells cotransfected with plasmid expressing SIRT3 or HBx were treated with a series concentration of peroxidant tBOOH. MTS assay showed that the HBx expression resulted in markedly downregulation of cell viability in response to tBOOH treatment, whereas ectopic expression of SIRT3 caused resistance of HBx-expressing cells to tBOOH treatment (Fig 4G). On the other hand, under the condition of oxidative stress, the apoptosis disparity in cells transfected different plasmids was detected by flow cytometry. As shown in Fig 4H, the percentage of apoptosis cells was significantly higher in the HBx expressing-cells than in the control cells. However, SIRT3 overexpression significantly abolished apoptosis induced by HBx. In conclusion, these data indicate that SIRT3 could protect cells expressing HBx from oxidant damage.

### SIRT3 attenuates increased HBV replication induced by ROS

**Fig 5:**
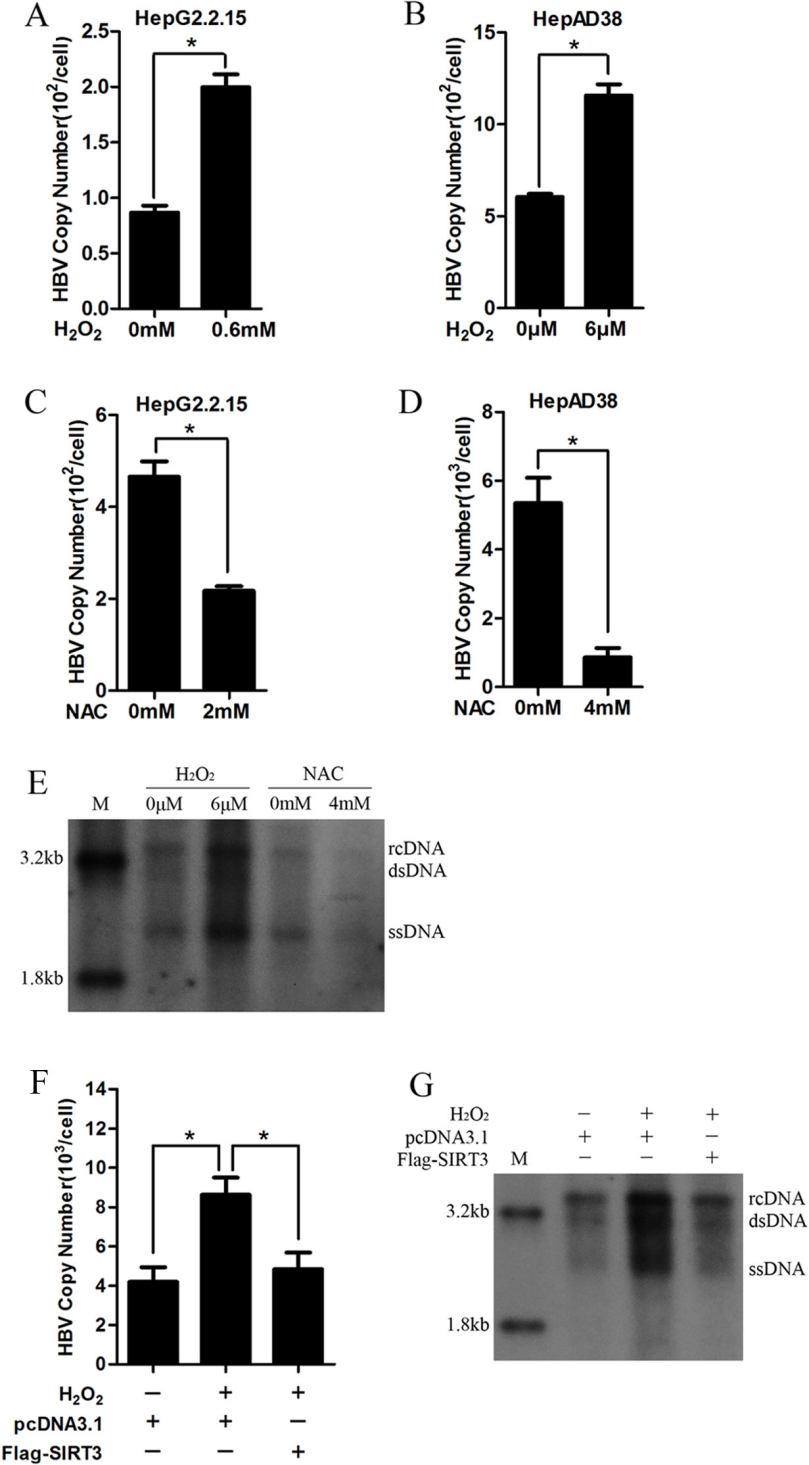
SIRT3 attenuates increased HBV replication induced by ROS. (A-B) HBV DNA replication intermediates in HepG2.2.15 (A) and HepAD38 cells (B) treated with H_2_O_2_ were analyzed by real-time PCR. *, p<0.01. (C-D) HBV DNA replication intermediates in HepG2.2.15 (C) and HepAD38 cells (D) treated with NAC were analyzed by real-time PCR. *, p<0.01. (E) HBV replication intermediates in HepAD38 cells treated with H_2_O_2_ and NAC were subjected to southern blot. (F-G) HBV DNA intermediates in Huh-7 cells overexpressing SIRT3 was analyzed by real-time PCR (F) and Southern blot (G). *, p<0.01. M, ladder; RC, intracellular HBV relax circle; DS, double strand DNA; SS, single strand DNA.

ROS could activate cellular signal pathways to modulate gene expression, cell adhesion, cell metabolism, cell cycle and cell death. To investigate the function of ROS in HBV replication, we treated two HBV stably expressing cell lines including HepG2.2.15 and HepAD38 with H_2_O_2_. Real-time PCR showed that H_2_O_2_ treatment increased HBV DNA replicative intermediates significantly (Fig 5A and 5B). In contrast, the treatment with antioxidant NAC inhibited HBV DNA replicative intermediates (Fig 5C and 5D). Moreover, the effect of H_2_O_2_ and NAC on HBV DNA replicative intermediates was further confirmed by southern blot (Fig 5E). These data suggest that cellular ROS could facilitate HBV replication. To elucidate the functional role of SIRT3 in the promotion of HBV replication induced by oxidative stress, HepG2.2.15 cells overexpressing SIRT3 was treated with H_2_O_2_. Ectopic expression of SIRT3 abolished enhancement of HBV DNA replicative intermediates induced by H_2_O_2_ both by real-time PCR and southern blot (Fig 5F and 5G). Taken together, these data reveal that SIRT3 decreases the promotion of oxidative stress to HBV replication.

## Discussion

Oxidative stress has been universally considered as a major contributor involved in pathological courses. It displays diverse oncogenic function, including the regulation of gene expression[14], epigenetic modification [15], cell death [16], genomic instability [17] as well as DNA mutation [18]. Several studies showed oxidative components were increased or antioxidants were decreased in either acute or chronic HBV infection [19–22]. In our study, we found that the HBV replication induced cellular ROS level based on different cell models, which suggests that HBV replication indeed induces oxidative stress.

Among the proteins encoded by the HBV genome, HBx is a key viral protein that plays an important role in oxidative stress. HBx could regulate fundamental aspects of mitochondrial physiology, which is a major source of reactive oxygen species (ROS). HBx protein interacts with a human voltage-dependent anion channel (HVDAC3) in the mitochondria and alters its transmembrane potential [23]. HBx also down-regulates mitochondrial enzymes involved in electron transport in oxidative phosphorylation (complexes I, III, IV, and V) and increases mitochondrial ROS level [24]. Moreover, mitochondrially associated HBx also leads to activation of transcription factors STAT-3 and NF-κB and mitochondrial translocation of Raf-1 via oxidative stress, which is relevant to liver disease pathogenesis [25, 26]. HBx expression is also associated with increased oxidative damage. Jung et al. reported that ROS production induced by the C-terminal region of HBx leaded to mitochondrial DNA damage, which might play a role in HCC development [27]. Consistently, our study first found the viral protein HBx played a major role in HBV-induced oxidative stress. HBx expression also caused the oxidatively induced DNA damage associated with ROS by measuring AP sites and γH_2_AX foci which is marker for double strand (DSB) DNA breaks. DNA damage provokes a rapid cellular response that either commits cells to repair the damaged DNA or leads cell to death. Our study also found that HBx expression resulted in increased percentage of apoptotic cell under peroxidant treatment. These data suggests HBx expression is associated with increased oxidative stress and DNA damage.

We further identified a novel cytoprotective factor, SIRT3, regulated the oxidative stress induced by HBx. There is a large number of evidence supporting the role of SIRT3 as a key regulator of oxidative stress and oxidative injury within cells. The deacetylase activity of SIRT3 results in a post-translational modification of mitochondrial proteome that directs intracellular oxidation-reduction pathways [28–30]. Recently, studies showed that several mitochondrial proteins, including members of oxidative phosphorylation chains [31], anti-oxidant factors [3], and metabolic associated proteins[32] were regulated by SIRT3. Cheng et al. revealed SIRT3 physically associated with OGG1 and prevented apoptotic cell death under oxidative stress [10]. Sundaresan et al. reported SIRT3 expression was necessary for cardiomyocytes to attenuate doxorubicin-induced mitochondrial ROS production [33]. Thereby, SIRT3 is uniquely poised to modulate both the ROS production and the detoxification of harmful free radicals, protecting cells from mitochondrial oxidative stress [3, 8, 34]. Indeed, we showed that overexpression of SIRT3 diminished ROS production induced by HBx expression, and restored GSH/GSSG which was unbalanced in HBx-expression cells. Importantly, SIRT3 overexpression markedly decreased oxidatively induced DNA damage associated with ROS in HBx-expressing cells. Consequently, ectopic expression of SIRT3 abolished cell death induced by HBx expression or peroxidant treatment.

Accumulating evidence suggested that cellular redox status played an important role in regulating viral replication and infectivity. Recently, Cai et al. reported that the thiol antioxidant GSH showed an anti-influenza activity *in vitro* and *in vivo* [35]. Aquaro et al. reported that antioxidant function of MnTBAP (a synthetic peroxynitrite decomposition catalyst) could modified the expression of virus proteins which were crucial for HIV-1 infection in both acutely and chronically infected monocytes/macrophages, suggesting that the removal of peroxynitrite contributed to the inhibition of HIV replication [36]. More Interesting, Enterovirus 71 infection induces ROS generation, which is required for viral replication [37]. In this study, we found H_2_O_2_ treatment significantly enhanced HBV replication, whereas the antioxidant NAC remarkably reduced HBV replication. SIRT3 overexpression attenuated the H_2_O_2_-induced HBV replication. It is undisputable that ROS generation has functional consequence on viral infection. In HBV, increased ROS not only facilitated HBV replication, but also caused oxidative damage to hepatocyte. These data provide evidence to prove the significance of antioxidant therapy in combination with antiviral therapy in HBV infection.

In summary, our study confirms that HBV/HBx could directly induce cellular oxidative stress and oxidative damage. SIRT3 overexpression could decrease ROS induct by HBx. Moreover, SIRT3 overexpression also markedly decreases HBx-induced oxidative stress and antagonizes HBV replication induced by oxidative stress.

## Supporting Information

S1 Fig SIRT3 attenuates the mitochondrial ROS level induced by HBx expression. (A-B) Mitochondrial ROS level in Huh-7 cells transfected with indicated plasmids were examined by MitoSOX^TM^ Red fluorescence (A) or flow cytometry (B). Magnification, ×200. *, p<0.05.
(TIF)

S2 Fig PRDX1 overexpression has no effect on HBx-induced ROS level. (A) Western blot analyzed HBx and PRDX1 in cells transfected with indicated plasmids. β-actin was used as a loading control. (B-C) ROS levels in Huh-7 cells transfected with indicated plasmids were examined by carboxy-H2DCFDA fluorescence (B) or flow cytometry (C). Magnification, ×100. *, p<0.01.
(TIF)

## References

[1] Neuveut C, Wei Y, Buendia MA. Mechanisms of HBV-related hepatocarcinogenesis. J Hepatol. 2010;52(4):594–604. Epub 2010/02/27. 10.1016/j.jhep.2009.10.033 . 20185200

[2] Lombard DB, Alt FW, Cheng HL, Bunkenborg J, Streeper RS, Mostoslavsky R, et al Mammalian Sir2 homolog SIRT3 regulates global mitochondrial lysine acetylation. Mol Cell Biol. 2007;27(24):8807–14. 10.1128/MCB.01636-07 17923681 PMC2169418

[3] Chen Y, Zhang J, Lin Y, Lei Q, Guan KL, Zhao S, et al Tumour suppressor SIRT3 deacetylates and activates manganese superoxide dismutase to scavenge ROS. EMBO Rep. 2011;12(6):534–41. 10.1038/embor.2011.65 21566644 PMC3128277

[4] Tao R, Coleman MC, Pennington JD, Ozden O, Park SH, Jiang H, et al Sirt3-mediated deacetylation of evolutionarily conserved lysine 122 regulates MnSOD activity in response to stress. Mol Cell. 2010;40(6):893–904. 10.1016/j.molcel.2010.12.013 21172655 PMC3266626

[5] Finley LW, Haas W, Desquiret-Dumas V, Wallace DC, Procaccio V, Gygi SP, et al Succinate dehydrogenase is a direct target of sirtuin 3 deacetylase activity. PLoS One. 2011;6(8):e23295 Epub 2011/08/23. 10.1371/journal.pone.0023295 21858060 PMC3157345

[6] Hirschey MD, Shimazu T, Goetzman E, Jing E, Schwer B, Lombard DB, et al SIRT3 regulates mitochondrial fatty-acid oxidation by reversible enzyme deacetylation. Nature. 2010;464(7285):121–5. 10.1038/nature08778 20203611 PMC2841477

[7] Pillai VB, Sundaresan NR, Kim G, Gupta M, Rajamohan SB, Pillai JB, et al Exogenous NAD blocks cardiac hypertrophic response via activation of the SIRT3-LKB1-AMP-activated kinase pathway. The Journal of biological chemistry. 2010;285(5):3133–44. 10.1074/jbc.M109.077271 19940131 PMC2823454

[8] Bause AS, Haigis MC. SIRT3 regulation of mitochondrial oxidative stress. Exp Gerontol. 2013;48(7):634–9. 10.1016/j.exger.2012.08.007 . 22964489

[9] Shulyakova N, Sidorova-Darmos E, Fong J, Zhang G, Mills LR, Eubanks JH. Over-expression of the Sirt3 sirtuin Protects neuronally differentiated PC12 Cells from degeneration induced by oxidative stress and trophic withdrawal. Brain Res. 2014;1587:40–53. 10.1016/j.brainres.2014.08.066 . 25194924

[10] Cheng Y, Ren X, Gowda AS, Shan Y, Zhang L, Yuan YS, et al Interaction of Sirt3 with OGG1 contributes to repair of mitochondrial DNA and protects from apoptotic cell death under oxidative stress. Cell Death Dis. 2013;4:e731 10.1038/cddis.2013.254 23868064 PMC3730425

[11] Hu L, Chen L, Yang G, Li L, Sun H, Chang Y, et al HBx sensitizes cells to oxidative stress-induced apoptosis by accelerating the loss of Mcl-1 protein via caspase-3 cascade. Mol Cancer. 2011;10:43 10.1186/1476-4598-10-43 21504623 PMC3096594

[12] Bolukbas C, Bolukbas FF, Horoz M, Aslan M, Celik H, Erel O. Increased oxidative stress associated with the severity of the liver disease in various forms of hepatitis B virus infection. BMC infectious diseases. 2005;5:95 10.1186/1471-2334-5-95 16262897 PMC1283973

[13] Chen CJ, Fu YC, Yu W, Wang W. SIRT3 protects cardiomyocytes from oxidative stress-mediated cell death by activating NF-kappaB. Biochem Biophys Res Commun. 2013;430(2):798–803. 10.1016/j.bbrc.2012.11.066 . 23201401

[14] Crow MT, Mani K, Nam YJ, Kitsis RN. The mitochondrial death pathway and cardiac myocyte apoptosis. Circ Res. 2004;95(10):957–70. 10.1161/01.RES.0000148632.35500.d9 . 15539639

[15] Lim SO, Gu JM, Kim MS, Kim HS, Park YN, Park CK, et al Epigenetic changes induced by reactive oxygen species in hepatocellular carcinoma: methylation of the E-cadherin promoter. Gastroenterology. 2008;135(6):2128–40, 40 e1-8. Epub 2008/09/20. 10.1053/j.gastro.2008.07.027 . 18801366

[16] Sakurai T, He G, Matsuzawa A, Yu GY, Maeda S, Hardiman G, et al Hepatocyte necrosis induced by oxidative stress and IL-1 alpha release mediate carcinogen-induced compensatory proliferation and liver tumorigenesis. Cancer Cell. 2008;14(2):156–65. Epub 2008/08/12. 10.1016/j.ccr.2008.06.016 18691550 PMC2707922

[17] Woo RA, Poon RY. Activated oncogenes promote and cooperate with chromosomal instability for neoplastic transformation. Genes Dev. 2004;18(11):1317–30. Epub 2004/06/04. 10.1101/gad.1165204 15175263 PMC420357

[18] Toyokuni S. Novel aspects of oxidative stress-associated carcinogenesis. Antioxid Redox Signal. 2006;8(7–8):1373–7. Epub 2006/08/17. 10.1089/ars.2006.8.1373 . 16910784

[19] Swietek K, Juszczyk J. Reduced glutathione concentration in erythrocytes of patients with acute and chronic viral hepatitis. Journal of viral hepatitis. 1997;4(2):139–41. Epub 1997/03/01. . 9097271 10.1111/j.1365-2893.1997.tb00217.x

[20] Chrobot AM, Szaflarska-Szczepanik A, Drewa G. Antioxidant defense in children with chronic viral hepatitis B and C. Medical science monitor: international medical journal of experimental and clinical research. 2000;6(4):713–8. Epub 2001/02/24. . 11208397

[21] Demirdag K, Yilmaz S, Ozdarendeli A, Ozden M, Kalkan A, Kilic SS. Levels of plasma malondialdehyde and erythrocyte antioxidant enzyme activities in patients with chronic hepatitis B. Hepato-gastroenterology. 2003;50(51):766–70. Epub 2003/06/28. . 12828081

[22] Irshad M, Chaudhuri PS, Joshi YK. Superoxide dismutase and total anti-oxidant levels in various forms of liver diseases. Hepatology research: the official journal of the Japan Society of Hepatology. 2002;23(3):178–84. Epub 2002/06/22. . 12076713 10.1016/s1386-6346(01)00181-4

[23] Rahmani Z, Huh KW, Lasher R, Siddiqui A. Hepatitis B virus X protein colocalizes to mitochondria with a human voltage-dependent anion channel, HVDAC3, and alters its transmembrane potential. Journal of virology. 2000;74(6):2840–6. Epub 2000/02/23. ; PubMed Central PMCID: PMCPmc111774. 10684300 10.1128/jvi.74.6.2840-2846.2000 PMC111774

[24] Lee YI, Hwang JM, Im JH, Lee YI, Kim NS, Kim DG, et al Human hepatitis B virus-X protein alters mitochondrial function and physiology in human liver cells. The Journal of biological chemistry. 2004;279(15):15460–71. 10.1074/jbc.M309280200 . 14724286

[25] Waris G, Huh KW, Siddiqui A. Mitochondrially associated hepatitis B virus X protein constitutively activates transcription factors STAT-3 and NF-kappa B via oxidative stress. Mol Cell Biol. 2001;21(22):7721–30. 10.1128/MCB.21.22.7721-7730.2001 11604508 PMC99943

[26] Chen J, Siddiqui A. Hepatitis B virus X protein stimulates the mitochondrial translocation of Raf-1 via oxidative stress. Journal of virology. 2007;81(12):6757–60. 10.1128/JVI.00172-07 17428866 PMC1900104

[27] Jung SY, Kim YJ. C-terminal region of HBx is crucial for mitochondrial DNA damage. Cancer Lett. 2013;331(1):76–83. Epub 2012/12/19. 10.1016/j.canlet.2012.12.004 . 23246371

[28] Guarente L. Mitochondria—a nexus for aging, calorie restriction, and sirtuins? Cell. 2008;132(2):171–6. Epub 2008/02/05. 10.1016/j.cell.2008.01.007 18243090 PMC2680180

[29] Smith KT, Workman JL. Introducing the acetylome. Nat Biotechnol. 2009;27(10):917–9. Epub 2009/10/10. 10.1038/nbt1009-917 . 19816449

[30] Hebert AS, Dittenhafer-Reed KE, Yu W, Bailey DJ, Selen ES, Boersma MD, et al Calorie restriction and SIRT3 trigger global reprogramming of the mitochondrial protein acetylome. Mol Cell. 2013;49(1):186–99. Epub 2012/12/04. 10.1016/j.molcel.2012.10.024 23201123 PMC3704155

[31] Cimen H, Han MJ, Yang Y, Tong Q, Koc H, Koc EC. Regulation of succinate dehydrogenase activity by SIRT3 in mammalian mitochondria. Biochemistry. 2010;49(2):304–11. Epub 2009/12/17. 10.1021/bi901627u 20000467 PMC2826167

[32] Hallows WC, Yu W, Smith BC, Devries MK, Ellinger JJ, Someya S, et al Sirt3 promotes the urea cycle and fatty acid oxidation during dietary restriction. Mol Cell. 2011;41(2):139–49. Epub 2011/01/25. 10.1016/j.molcel.2011.01.002 21255725 PMC3101115

[33] Sundaresan NR, Samant SA, Pillai VB, Rajamohan SB, Gupta MP. SIRT3 is a stress-responsive deacetylase in cardiomyocytes that protects cells from stress-mediated cell death by deacetylation of Ku70. Mol Cell Biol. 2008;28(20):6384–401. Epub 2008/08/20. 10.1128/mcb.00426-08 18710944 PMC2577434

[34] Yu W, Dittenhafer-Reed KE, Denu JM. SIRT3 protein deacetylates isocitrate dehydrogenase 2 (IDH2) and regulates mitochondrial redox status. The Journal of biological chemistry. 2012;287(17):14078–86. Epub 2012/03/15. 10.1074/jbc.M112.355206 22416140 PMC3340192

[35] Cai J, Chen Y, Seth S, Furukawa S, Compans RW, Jones DP. Inhibition of influenza infection by glutathione. Free Radic Biol Med. 2003;34(7):928–36. Epub 2003/03/26. . 12654482 10.1016/s0891-5849(03)00023-6

[36] Aquaro S, Muscoli C, Ranazzi A, Pollicita M, Granato T, Masuelli L, et al The contribution of peroxynitrite generation in HIV replication in human primary macrophages. Retrovirology. 2007;4:76 10.1186/1742-4690-4-76 17949509 PMC2173904

[37] Cheng ML, Weng SF, Kuo CH, Ho HY. Enterovirus 71 induces mitochondrial reactive oxygen species generation that is required for efficient replication. PLoS One. 2014;9(11):e113234 Epub 2014/11/18. 10.1371/journal.pone.0113234 25401329 PMC4234665

