# Supplementary figures and images for: Protective Role of Sirtuin3 (SIRT3) in Oxidative Stress Mediated by Hepatitis B Virus X Protein Expression
Source: PLoS One. 2016 Mar 7;11(3):e0150961. doi: 10.1371/journal.pone.0150961 (PMC4780820; doi:10.1371/journal.pone.0150961)

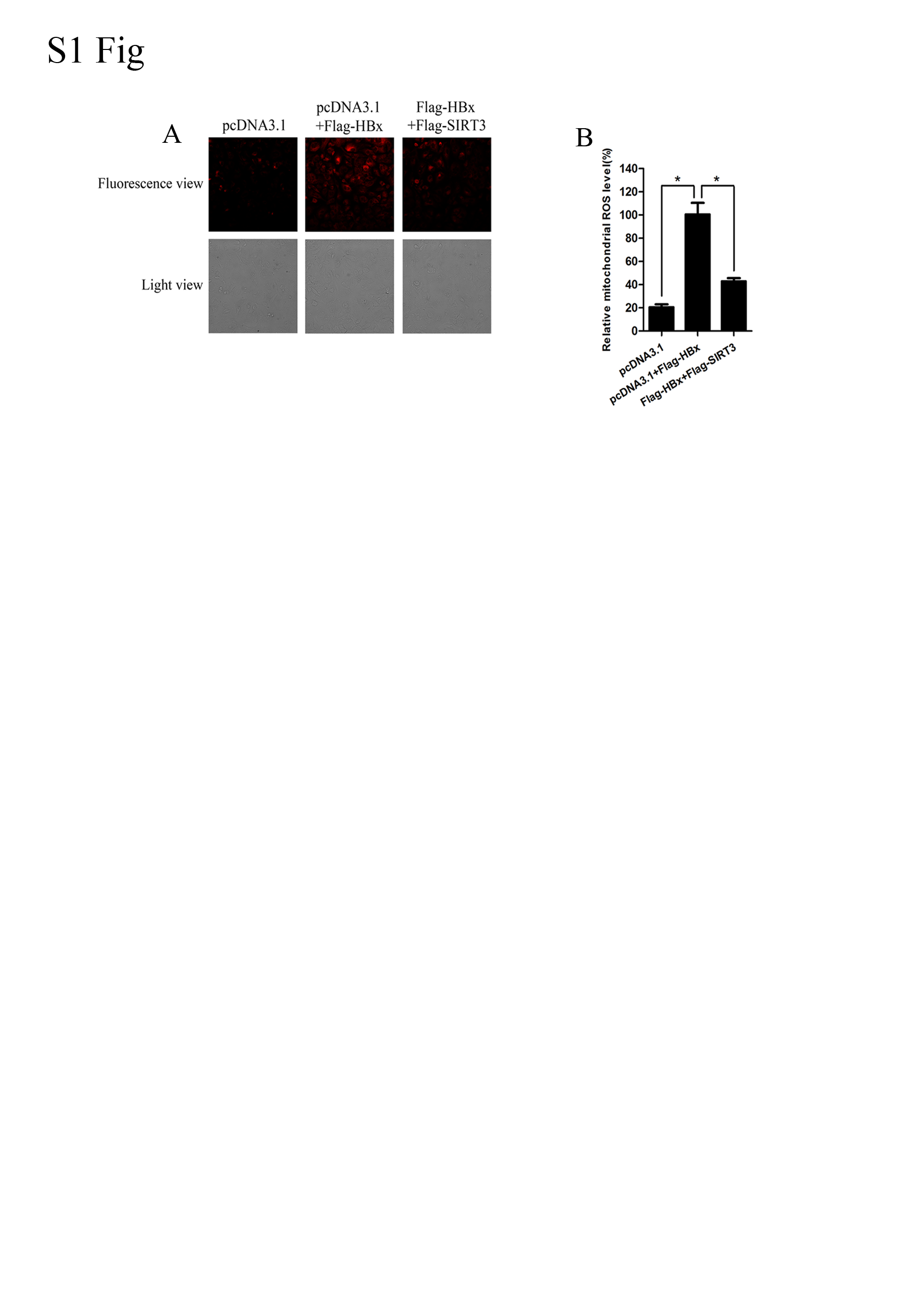

Supplement: S1 Fig — (A-B) Mitochondrial ROS level in Huh-7 cells transfected with indicated plasmids were examined by MitoSOXTM Red fluorescence (A) or flow cytometry (B). Magnification, ×200. *, p<0.05. (TIF) [file pone.0150961.s001.tif]

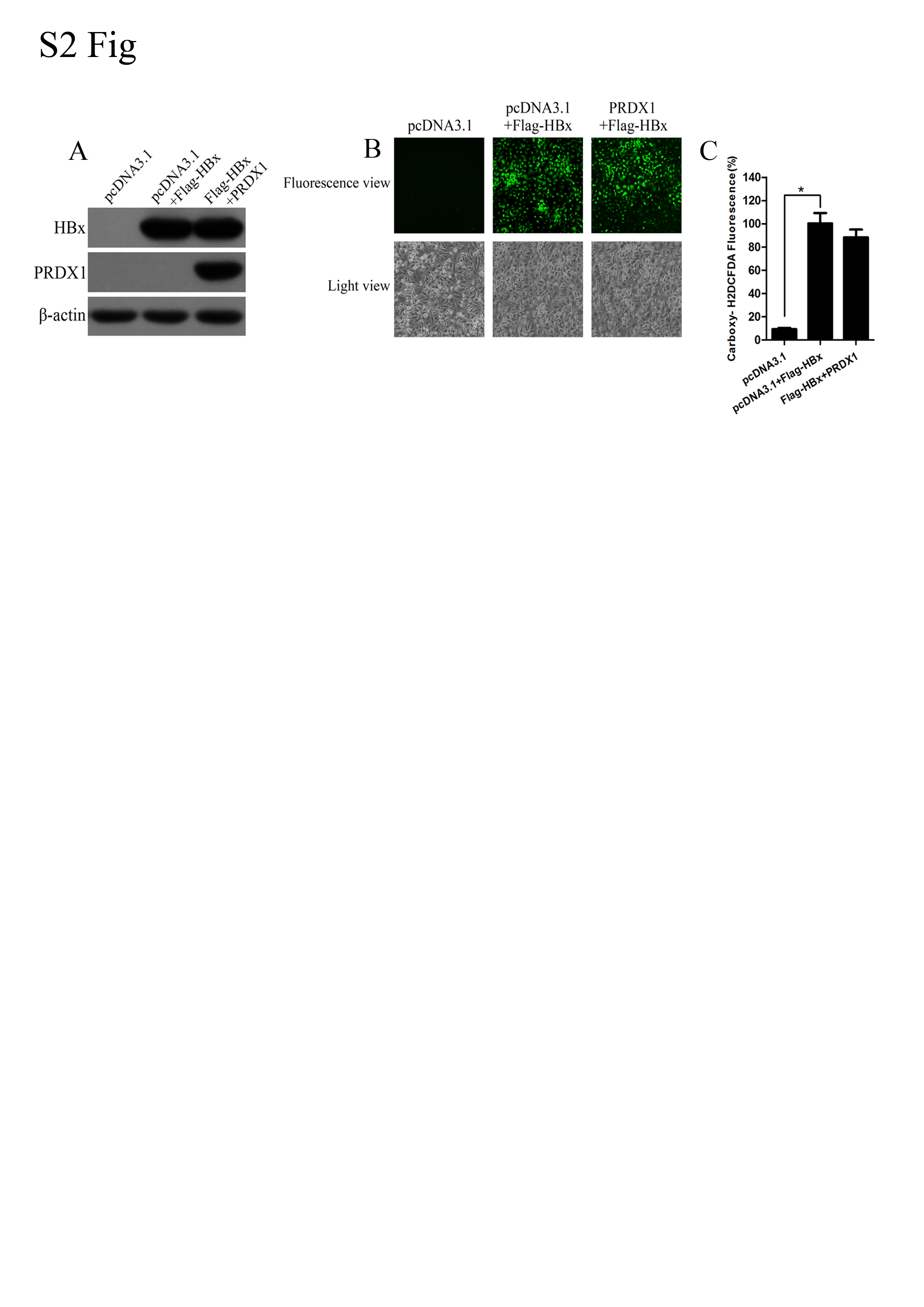

Supplement: S2 Fig — (A) Western blot analyzed HBx and PRDX1 in cells transfected with indicated plasmids. β-actin was used as a loading control. (B-C) ROS levels in Huh-7 cells transfected with indicated plasmids were examined by carboxy-H2DCFDA fluorescence (B) or flow cytometry (C). Magnification, ×100. *, p<0.01. (TIF) [file pone.0150961.s002.tif]
